# Effect of the posterior stop on temporomandibular disorders: A systematic review

**DOI:** 10.34172/joddd.2022.025

**Published:** 2022-11-15

**Authors:** Zahra Jamali, Negin Hadilou, Amin Nourizadeh

**Affiliations:** ^1^Department of Oral Medicine, Faculty of Dentistry, Tabriz University of Medical Sciences, Tabriz, Iran; ^2^Faculty of Dentistry, Tabriz University of Medical Sciences, Tabriz, Iran; ^3^Department of Prosthodontics, Faculty of Dentistry, Tabriz University of Medical Sciences, Tabriz, Iran

**Keywords:** Dental occlusion, Posterior missing teeth, Prosthodontics, Temporomandibular disorders

## Abstract

**Background.** Temporomandibular disorders have common signs and symptoms, including pain in the masticatory muscles, limitation or deviation in a mandibular range of motion, and other common patient complaints, such as headache and earache. The main focus of this study was to collect comprehensive and integrated data on the effect of the posterior stop on temporomandibular joint disorders, as well as prevention, treatment, and follow-up care for the patients.

**Methods.** The authors conducted the search in PubMed, SCOPUS, Web of Science, Cochrane Library, CINHAL, Medline, ProQuest, Google Scholar, Magiran, IranDoc, SID, and Iranmedex databases for relevant articles. A list of selected study sources, related conferences, and grey literature were manually searched in addition to the databases mentioned above. A 21-year time limit was imposed (2000-2021).

**Results.** Finally, 16 articles were selected to be reviewed in this systematic review. The designs of the included studies were heterogeneous, and due to the low number of studies covered, the authors could not carry out a meta-analysis.

**Conclusion.** The causes of temporomandibular disorders are multifactorial and complex. Therefore, it is difficult to investigate the relationship between this disorder and predictors. The results of the present study indicate that to determine the effect of the posterior stop on temporomandibular joint disorders, more clinical trials and case-control studies should be conducted.

## Introduction

 Temporomandibular joint disorder (TMD) is a catch-all term for a variety of clinical issues involving the masticatory muscle, the temporomandibular joints (TMJs), and adjacent tissues.^1^ Temporomandibular disorders have common signs and symptoms.^2^ Symptoms include pain in the masticatory muscles or the TMJ, limitation or deviation in mandibular range of motion, TMJ noise during mandibular activity, and other common patient complaints such as headache and earache.^3,4^ TMD was initially studied scientifically in the 1950s.^2^ The cause of this condition and treatment choices are still subject to debate.^5,6^ TMD problems have a complex etiology that has only been partially identified. They relate to factors like malocclusion, trauma, mental stress (bruxism and clenching), and chewing muscle activity.^7,8^ The most prevalent complaint associated with TMD is masticatory muscle dysfunction.^2-4^ The two types of chewing muscle activities related to TMD are functional such as chewing, and parafunctional, such as clenching the teeth.^9^ According to preliminary scientific studies, Occlusal factors can affect the function of masticatory muscles.^2^ In addition, the relationship between missing posterior teeth and TMD is unknown.^10^ Some studies have found a relationship between occlusal variables and masticatory muscle pain, whereas others have found no such relation.^1,2^

 Several studies have linked tooth loss and occlusal abnormalities to the occurrence of TMD.^7,11^ The incidence of temporomandibular disorders appears to be significantly reduced when occlusion is controlled.^12^ TMD symptoms and signs are related to posterior tooth loss.^2,13^ Using MRI, Tallents et al^6^ showed a real relationship between mandibular posterior tooth loss and TMD. In the same vein, the results of some other studies, including a study by Wang et al,^14^ showed that loss of posterior teeth in more than one quadrant causes TMD to be more prevalent, especially in young people.

 However, some research has found no relationship between TMD, occlusion, and missing posterior teeth.^15-19^ A study by Manfredini et al^17^ did not confirm a clinical relationship between TMD and occlusion. According to Witter et al,^18^ the shorter dental arch provides occlusal stability, and the free end partial denture does not prevent TMD or improve patient performance. Prosthodontic therapy has also been found to be ineffective in treating TMD.^20^ TMD cannot be cured because of the long-term effects of missing teeth on jaw function, even when missing posterior teeth are replaced with dentures.^6^

 Recent studies have shown that occlusal treatment has only short-term benefits. Occlusal grinding or restoration cannot be distinguished from conservative and basic procedures such as occlusal applause or physical therapy because of a lack of evidence. Many studies, however, have shown that occlusal therapy is effective in rehabilitating patients with TMD. Other studies have similarly found little benefit from using a removable or fixed prosthesis to treat TMD, especially if the patient has just lost their molar teeth. In some situations, however, it may be necessary to replace the lost teeth to stabilize the device.^10^

 Further studies should be performed to investigate the long-term effects of occlusal changes and biomechanical instability on masticatory system disorders and TMD.^10^

 Given the conflicting results in previous studies, this study sought to gather complete and integrated information on the effect of the posterior stop on TMDs, prevention, treatment, and follow-up care for the patients.

## Methods

###  Search strategy

 In this systematic review, the available evidence and previous studies on the effect of the posterior stop on TMDs were investigated.

 Articles were searched in PubMed, SCOPUS, Web of Sciences, Cochrane Library, CINHAL, Medline, ProQuest, Google Scholar, and other valid databases. Regarding Persian databases, Magiran, IranDoc, SID, and Iranmedex were searched. A list of selected study sources, as well as related conferences and grey literature, were manually searched in addition to the databases mentioned above.

 Articles were selected based on their titles, abstracts, and references used.

 Using the PEO framework, the following keywords and search strings were selected to make the final selection of articles:

 P (Population/Patient/ Problem): (MeSH Terms) temporomandibular disorder, tooth loss, dental occlusion

 E (Exposure): (MeSH Terms) prosthodontic, implant

 O (Outcomes): (MeSH Terms) pain, mandibular range of motion, patient satisfaction

 Keywords used to search for related articles were temporomandibular disorder, tooth loss, posterior missing teeth, dental occlusion, prosthodontic, pain, and patient satisfaction. The keywords were searched as MeSH terms and free terms.

###  Inclusion and exclusion criteria

 Inclusion criteria for all articles included in this study were:

 They were published between 2000 and 2021, in English or Persian, and used specific and related work methods; they were clinical trials and in vivo studies and studied the correlation between the posterior stop and TMD.

 Review articles, in vitro and animal articles, case reports, letters, questionnaires, and low-quality publications were excluded. The study also excluded studies that entirely focused on rheumatoid arthritis and joint and periodontal ligament structural abnormalities.

###  Data extraction

 In three steps, two reviewers screened the studies. Duplicate articles were discovered and removed first. The titles and abstracts were then reviewed to see if they met the eligibility criteria. Following that, the full texts of articles that met the eligibility requirements were selected. If the two reviewers could not agree on whether or not the study should be included, a third reviewer was asked to decide.

 Endnote 20 resource management software was used to organize titles and abstracts and identify duplicates. The JBI (Joanna Briggs Institute)^21^ checklists were used to assess the risk of bias.

## Results

**Figure 1:**
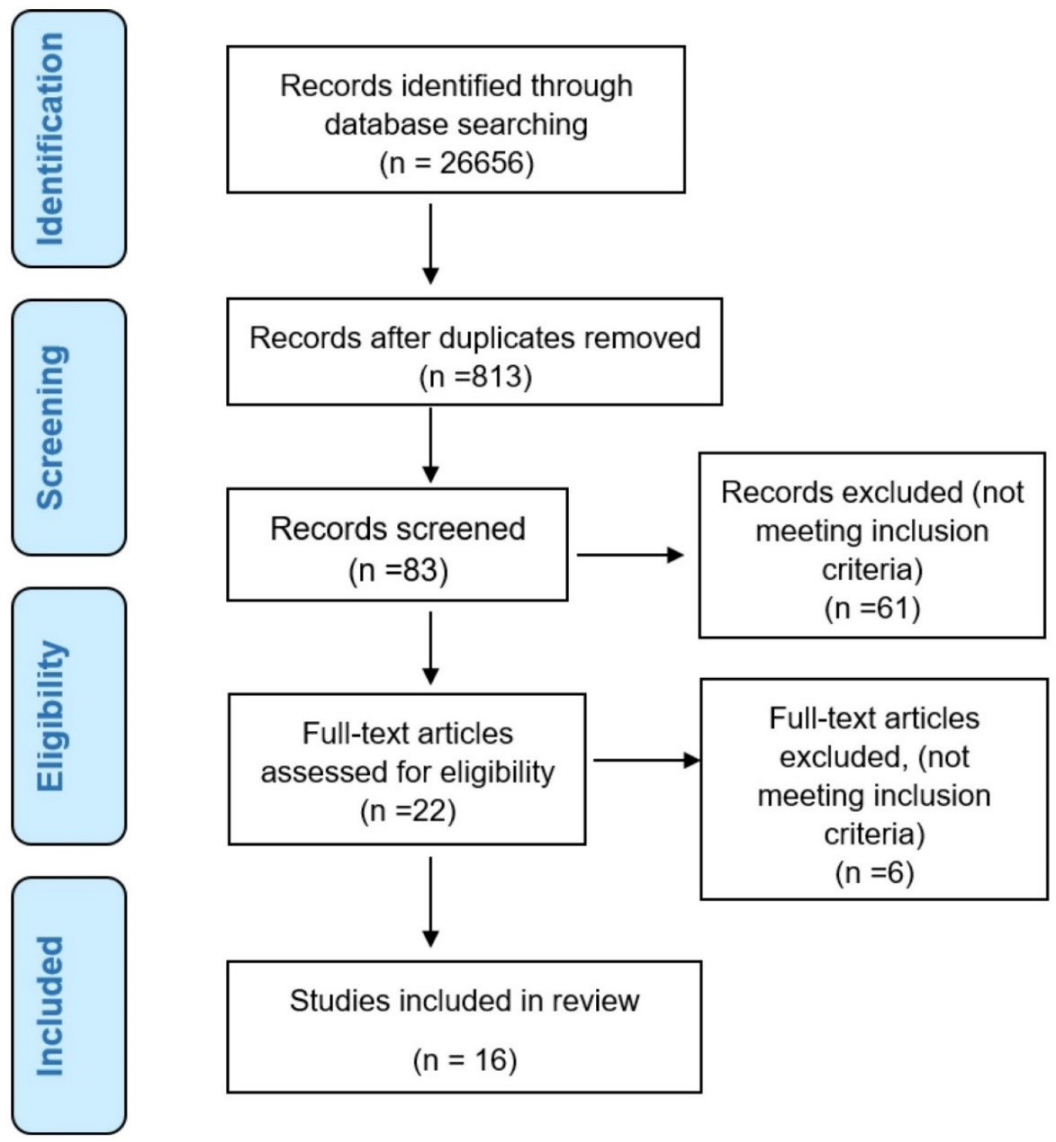
Flow diagram (PRISMA)

 Initially, 26 656 studies were found in the search. After removing the duplicate articles and rescreening the titles and abstracts, all the articles that did not fulfill the review’s eligibility criteria were excluded. Twenty-two full-text papers were selected for the screening, of which six papers were excluded because of predetermined criteria and the study’s objectives. In the end, sixteen studies were chosen (Table 1). Figure 1 shows the flow diagram of the study selection process and results of the literature search according to PRISMA guidelines.^22^

**Table 1:** Qualitative information

**Author (year)**	**Study type**	**Variables**	**Results**
Tallents et al^6^ (2002)	Comparative study	- Missing posterior teeth - Disk displacement (DD)	There is a connection between lost mandibular posterior teeth and disk displacement.
Vojdani and Asadi^24^ (2003)	Cross-sectional epidemiologic	TMJ malformation	Tooth loss is clearly connected to temporomandibular malformation.
Witter et al^32^ (2007)	Observational cohort study	Signs and symptoms of TMD	There was no significant difference in signs and symptoms related to temporomandibular disorders between the groups.
Wang et al^10^ (2007)	Comparative study	Gender, age, number of missing teeth, TLO, joint symmetry, and osseous changes	Some signs and symptoms of TMD are related to a tightly locked occlusion.
Schmitter et al^29^ (2007)	Case series	Myofascial pain and occlusal factors	Myofascial pain is more likely when there is an open bite and no posterior occlusion.
Wang et al^14^ (2009)	Descriptive and analytical	Gender, age, number of missing posterior teeth, number of dental quarters with missing posterior teeth	TMD is more likely if there are more quadrants with missing posterior teeth. The female gender and young age both enhance the chance of TMD development.
Haralur et al^28^ (2014)	Random cross-sectional	Malocclusion, occlusal interference, and TMD	Anterior open bite, incisal spacing, and increased max overjet were strongly linked with TMD.
Reissmann et al^31^ (2014)	Randomized controlled trial	Pain intensity (TMD)	Compared to no tooth replacement, tooth replacement (RDP) did not affect the risk of TMD. TMD is not caused by SDA.
de Sousa et al^26^ (2015)	Case series	Correlation between malocclusion and tooth loss and TMD	There was no link between TMD, occlusal factors, and tooth loss in this study.
Prithi and Pradeep^8^ (2016)	Descriptive and analytical	clicking sounds, pain, side of chewing preference, number of missing teeth	The number of missing posterior teeth increased with age and was linked to TMD symptoms and signs.
Jahandideh et al^25^ (2017)	Descriptive and cross-sectional	Signs and symptoms of TMD	TMD development can be influenced by factors such as age, parafunctional habits, trauma, significant attrition, jaw dislocation, loss of posterior teeth, and financial status.
Nguyen et al^11^ (2017)	Cross-sectional	Signs and symptoms of TMD	The loss of all OSZs on one or both sides of the mouth was a predictor of TMD.
Manchikalapudi and Polasani^30^ (2017)	Case-control study	Signs and symptoms of TMD	In terms of their association with TMD signs and symptoms, statistically, there was no significant difference between the groups.
de Lourdes Sá de Lira and Vasconcelos Fontenele^27^ (2020)	A cross-sectional, quantitative, non-randomized clinical interventional study	- Malocclusion, - Tooth loss	There was no link between malocclusion and tooth loss and TMD symptoms and signs.
Omrani et al^23^ (2021)	Descriptive and analytical	Signs and symptoms of TMD	Posterior edentulism, earache, bruxism, a history of jaw dislocation, headache, and migraine are associated with temporomandibular disorders.
Emshoff et al^33^ (2021)	Retrospective paired-design study	- Missing posterior teeth - Arthralgia (MRI findings) - Disk displacement without reduction - Bone marrow edema	Missing posterior teeth, TMJ arthralgia, disk displacement without reduction, and bone marrow edema were predictors of condylar erosion.

DD, Disk displacement; TMD, Temporomandibular disorder; TLO, Tightly locked occlusion; RDP, Removable dental prosthese; SDA, Shortened dental arches; OSZ, Occlusal support zone; MRI, Magnetic resonance images; TMJ, Temporomandibular joint.

 No systematic review and review study has been conducted concerning the purpose of the research.

 The JBI checklists^21^ were used to assess the risk of bias. This checklist has four alternatives for each question: Yes, No, Unclear, and Not Applicable. Based on the obtained scores, the articles were rated as strong (scored>75%), medium (scored>50%), and weak (scored<50%). The study has a high risk of bias because of the articles without controlled clinical trials or the ones which were mainly descriptive with no control, matching, or randomization groups.

 The design of the included studies was heterogeneous, and due to the low number of studies covered, the authors could not perform a meta-analysis.

## Discussion

 The TMD is characterized by pain in the jaw joint area and around the ear and, in some cases, recurrent pain in the TMJ, neck, or shoulders, TMJ sounds during movement, limitation in opening the jaw, and deviation.^1,3^ Crepitus, clicking, increased contraction of the masticatory muscles, pain in the TMJ joint, headache, and limitation of jaw motions are the most typical symptoms of TMD.^4-6^ Posterior tooth loss reduces chewing efficiency, causes posterior abutment loss and movement of remaining teeth, and changes occlusal contacts, raising the risk of temporomandibular disorders (TMD).^6^

###  Posterior tooth loss and TMD

 The relationship between posterior tooth loss and the development of TMD has been widely studied, and edentulism, particularly the absence of posterior teeth, has been linked to temporomandibular disorders.^6,8,23,24^

 Tallents et al^6^ divided their subjects into four groups with and without TMD and with and without disc displacement (based on magnetic resonance imaging) and observed that in addition to a positive correlation with disc replacement, the loss of mandibular teeth is also associated with the progression of the degenerative joint disease. Similarly, Prithi and Pradeep^8^ observed a correlation between signs and symptoms of TMD and the loss of posterior teeth and a relationship between TMD and aging. In an investigation of factors connected to temporomandibular disorders, Omrani et al^23^ identified the loss of posterior teeth as the most effective factor. In assessing the prevalence of TMJ disease and related factors, Jahandideh et al^25^ identified the loss of posterior teeth as a cause of TMD. Vojdani and Asadi^24^ discovered connections between the intensity of TMJ malformation and tooth loss. However, de Sousa et al^26^ reported contradictory findings as no correlation between TMD and tooth loss was found in their study.

 Wang et al^14^ provided more details. The number of quadrants with missing posterior teeth increased the likelihood of TMD.^14^ Nguyen et al^11^ reported similar results in predicting the risk of TMD in the elderly with loss of occlusal support zones on one or both sides of the patients’ mouth. However, a study by Manfredini et al^17^ did not support the existence of a clinical relationship between TMD and occlusion.

 When teeth are lost, especially at a young age, the teeth/tooth usually deviate from their original vertical position, changing the angle of the surrounding teeth/tooth.^2^ According to a study by Wang et al,^14^ younger patients are more prone to TMD.

 On the other contrary, Schmidt-Kaunisaho et al^34^ found that the occurrence of TMJ malformation decreased with age and decreased tooth count. However, it has long been believed that the incidence of TMJ dysfunction rises with age and tooth loss. This might be because the likelihood of malformation or dysfunction in all body organs, including the joints and the TMJ, increases with age.^34^

 According to Carlsson’s epidemiological study, those with fewer teeth had higher symptoms of temporomandibular malignancy than those with more teeth.^35^ In other words, the number of teeth had an inverse relationship with the frequency and severity of TMJ dysfunction, and the symptoms of malignancy increased as the number of teeth decreased. This increase could be attributed to osteoarthritis affecting TMJs.^35^

###  TMD and Malocclusion 

 The relationship between malocclusion, TMD, and tooth loss has been studied in some research. De Lourdes Sá de Lira and Vasconcelos Fontenele et al^27^ found no significant relationship between malocclusion and tooth loss in patients with TMD symptoms while finding a strong relationship between tooth loss and all the symptoms of TMD. According to the study, patients who had lost numerous teeth and lacked malocclusion had higher signs and symptoms of TMD.^27^ De Sousa et al^26^ found no relationship between TMD and occlusal factors (anterior open bite, posterior crossbite, excessive overbite, and overjet elements).

 However, Haralur et al^28^ reported a relationship between malocclusion and missing teeth in the posterior teeth and a positive relationship between TMD and anterior open bite, increased maxillary overjet, and decreased occlusal contacts. Uncoordinated and asymmetric muscle activity, abnormal occlusion, and masticatory system imbalance result from the lack of bilateral symmetry in occlusal interactions.^28^ Schmitter et al^29^ highlighted the significance of occlusion. The presence of open bite and a lack of occlusion increased the likelihood of myofascial pain.

###  Comparing the symptoms of temporomandibular disorders

 Different outcomes have been found in studies comparing the symptoms of temporomandibular disorders in edentulous patients people and those with tooth replacement. In their study of the link between posterior edentulism and temporomandibular disorder, Manchikalapudi and Polasani^30^ found no statistically significant difference in the prevalence of TMD signs and symptoms between the posterior edentulous and prosthetic groups. According to Reissmann et al,^31^ tooth replacement (RDP) did not affect TMD risk compared to no tooth replacement (SDA). To put it another way, missing molars do not need to be replaced to prevent TMD pain.^31^

 Witter et al^32^ found no significant differences in signs and symptoms of temporomandibular disorders between individuals with a short dental arch and those with a complete dental arch. Also, according to Witter et al,^18^ a shortened dental arch provides occlusal stability, and the free-end partial denture does not prevent TMD or improve patient performance.

 According to Emshoff et al,^33^ in patients with TMJ arthralgia, missing posterior teeth effectively predict condyle erosion.

###  Prosthetic treatment

 According to some studies, prosthetic treatment in TMD patients is not appropriate for the initial treatment of TMD. These studies undervalue the influence of tooth loss and the lack of posterior occlusal support on TMD, casting doubts on the efficacy of prosthetic restorations as a TMD preventative or treatment option.^20^

## Conclusion

 According to most researchers, the causes of TMD are multifactorial and complex. Therefore, it is difficult to investigate the relationship between this disorder and predictors. Age, gender, parafunctional habits, and psychological and socioeconomic status are confounding variables and increase the risk of bias in the results if not controlled. Also, different criteria for determining temporomandibular disorders and different diagnostic methods (clinical, self-reported, and radiographic images) cause differences in the results of studies. Due to the limited number of studies on gender, it cannot be determined whether it impacts TMD.

 The present study indicated that clinical trials and case‒control studies should be performed to determine the effect of the posterior stop on TMDs.

## Suggestions

 Due to the limited number of clinical trials in this area, it is recommended that more research be undertaken based on the number of quadrants with missing posterior teeth and the influence of prosthetic and implant therapies on TMD signs and symptoms.

## Author Contributions

**Conceptualization:** Zahra Jamali.

**Methodology: **Zahra Jamali, Amin Nourizadeh.

**Validation: **Amin Nourizadeh.

**Investigation:** Negin Hadilou.

**Resources:** Zahra Jamali.

**Writing—Original Draft Preparation:** Negin Hadilou.

**Writing—Review and Editing:** Zahra Jamali, Amin Nourizadeh.

**Supervision: **Zahra Jamali.

**Project Administration: **Zahra Jamali.

## Funding

 Not applicable.

## Ethics Approval

 The study protocol was approved by the Ethics Committee of the Research Council of Tabriz Dental School, Tabriz, Iran (approval number: IR.TBZMED.VCR.REC.1400.214).

## Competing Interests

 The authors declare no competing interests with regard to the authorship and/or publication of this article.
